# Multi-institutional Analysis of the Clinical and Genomic Characteristics of Black Patients with Metastatic Hormone-Sensitive Prostate Cancer

**DOI:** 10.1093/oncolo/oyab057

**Published:** 2022-02-24

**Authors:** Meredith N Freeman, Albert Jang, Jason Zhu, Farhad Sanati, Lakshminarayanan Nandagopal, Deepak Ravindranathan, Arpita Desai, Audrey Phone, Roberto Nussenzveig, Ellen Jaeger, Sydney A Caputo, Vadim S Koshkin, Umang Swami, Arnab Basu, Mehmet A Bilen, Neeraj Agarwal, Oliver Sartor, Earle F Burgess, Pedro C Barata

**Affiliations:** Tulane Cancer Center, Tulane University School of Medicine, New Orleans, LA, USA; Tulane Cancer Center, Tulane University School of Medicine, New Orleans, LA, USA; Levine Cancer Institute, Atrium Health, Charlotte, NC, USA; University Hospitals Seidman Cancer Center, Cleveland, OH, USA; University of Alabama at Birmingham, Birmingham, AL, USA; Winship Cancer Institute of Emory University, Atlanta, GA, USA; University of California San Francisco, Helen Diller Family Comprehensive Cancer Center, San Francisco, CA, USA; University of California San Francisco, Helen Diller Family Comprehensive Cancer Center, San Francisco, CA, USA; Huntsman Cancer Institute-University of Utah Health Care, Salt Lake City, UT, USA; Tulane Cancer Center, Tulane University School of Medicine, New Orleans, LA, USA; Tulane Cancer Center, Tulane University School of Medicine, New Orleans, LA, USA; University of California San Francisco, Helen Diller Family Comprehensive Cancer Center, San Francisco, CA, USA; Huntsman Cancer Institute-University of Utah Health Care, Salt Lake City, UT, USA; University of Alabama at Birmingham, Birmingham, AL, USA; Winship Cancer Institute of Emory University, Atlanta, GA, USA; Huntsman Cancer Institute-University of Utah Health Care, Salt Lake City, UT, USA; Tulane Cancer Center, Tulane University School of Medicine, New Orleans, LA, USA; Levine Cancer Institute, Atrium Health, Charlotte, NC, USA; Tulane Cancer Center, Tulane University School of Medicine, New Orleans, LA, USA

**Keywords:** prostatic neoplasms, androgen deprivation therapy, health care disparities, African Americans, steroid synthesis inhibitors, DNA sequence analysis

## Abstract

**Background:**

The outcomes of metastatic hormone-sensitive prostate cancer (mHSPC) have significantly improved through treatment intensification, yet Black representation in those studies is suboptimal.

**Methods:**

A multi-institutional, retrospective analysis of Black men with mHSPC was conducted, focusing on baseline demographics, treatment patterns, genomic profiles, clinical outcomes including prostate-specific antigen response, time to castrate-resistant prostate cancer (CRPC), and subsequent treatments.

**Results:**

A total of 107 patients, median age 64 years, 62% with de novo metastases at diagnosis and 64% with high-volume disease, were included. Twenty-nine patients (27%) were treated with androgen deprivation therapy (ADT) with and without first generation anti-androgens, while 20%, 38% and 5% received chemotherapy, abiraterone, and enzalutamide, respectively. At time of data cut-off, 57 (54%) patients had developed CRPC, with a median time to CRPC of 25.4 months (95% CI 20.3-30.4). The median time to CRPC was 46.3 months (18.9-73.7) and 23.4 months (18.6-28.2) for patients who received ADT with or without first-generation anti-androgens and treatment intensification, respectively. The 2-year survival rate was 93.3%, and estimated median overall survival of was 74.9 months (95% CI, 68.7-81.0). Most patients (90%) underwent germline testing; the most frequent known alterations were found within the DNA repair group of genes. Somatic testing revealed pathogenic alterations of interest, notably *TP53* (24%) and *CDK12* (12%).

**Conclusion:**

In our cohort, Black men with mHSPC presented with a high proportion of de novo metastases and high-volume disease. Treatment outcomes were very favorable with ADT-based regimens. The genomic landscape suggests different molecular profile relative to White patients with potential therapeutic implications.

## Teaser

Racial disparities play a role in clinical trial enrollment, treatment implications, and clinical outcomes of patients with prostate cancer. This article reports on clinical and treatment outcomes and provides additional insights on the somatic and germline profile of Black men with metastatic hormone-sensitive prostate cancer.

**Implications for Practice**

Racial disparities unfortunately play a role in clinical trial enrollment, treatment implications, and clinical outcomes between Black and White patients with prostate cancer. In this multi-institutional study of Black patients with hormone-sensitive prostate cancer, the observed clinical outcomes were very favorable with androgen deprivation therapy-based regimens. The genomic testing revealed different germline and somatic mutations with significant impact in the management of these patients.

## Introduction

The incidence of metastatic prostate cancer in the United States has been rising, from 4% in 2003 to 8% in 2017.^1^ Approximately 7% of patients with prostate cancerpresent with de novo distant metastases,^2^ and up to 30% of patients initially diagnosed with and treated for localized prostate cancer develop metastases.^3,4^ With more treatment options now available, the 5-year survival rate of metastatic prostate cancer has slightly increased in recent decades but still hovers around 30%.^1^ Virtually all patients with metastatic disease initially respond to castration; however, progression is inevitable, leading to metastatic castrate-resistant prostate cancer (CRPC).

Until 2015, the standard treatment for metastatic hormone-sensitive prostate cancer (mHSPC) was based on castration alone^5,6^ or combined with first-generation anti-androgens.^7-9^ Since then, the standard of care has rapidly evolved to include novel hormonal therapies or chemotherapy based on the positive results from phase III trials. When combined with androgen deprivation therapy (ADT), docetaxel,^10,11^ abiraterone,^12,13^ enzalutamide,^14,15^ and apalutamide^16^ improved all efficacy endpoints irrespective of tumor volume. However, Black patient representation in these studies was very low, ranging from 1.4% to 9.6% of enrolled participants.^10,16-18^ This underrepresentation is ubiquitous across mainstream prostate cancer trials, as shown in an analysis of 72 global phases 3 and 4 trials involving prostate cancer, in which an astounding 809,978 of 844,002 participants (96.0%) with available race data were White.^19^

This discrepancy is more concerning given that compared with non-Hispanic White men, non-Hispanic Black men have a much higher chance of developing prostate cancer and dying from prostate cancer.^20^ Socioeconomic factors, access to health care, baseline medical conditions, and underlying genetic factors may all play a role. Nevertheless, Black men with low-risk prostate cancer were found to have a higher risk of dying compared to non-Black men with similar disease burden.^21^ Intriguingly, when comparing CRPC treatment outcomes by race, Black patients may have better outcomes with docetaxel,^22^ radium-223,^23^ and sipuleucel-T.^24^ The Black patient underrepresentation along with these paradoxical findings warrants further investigation. Recent advancements to improve the accessibility of germline and somatic sequencing provide a unique opportunity to evaluate the potential role of genetics in these disparities. Our group with collaborators has previously revealed differences in the tumor genomic profile of Black versus White men with advanced prostate cancer.^25,26^

This multi-institutional, retrospective study aimed to report clinical and treatment outcomes and provide additional insights on the somatic and germline profile of Black men with mHSPC.

## Methods

### Study Design and Patient Population

A retrospective study was conducted with data from 7 academic institutions across 7 states treating Black men with mHSPC, with year of stage 4 diagnosis ranging from 2008 to 2021 (with 87% of patients diagnosed with stage 4 disease in the year 2015 or later). Participants included male patients who self-identified as Black, with known mHSPC, for whom somatic and/or germline genome sequence data were available.

Patients were divided into 2 main treatment groups: ADT ± first-generation anti-androgen and treatment intensification. Treatment intensification included ADT plus chemotherapy and/or a novel hormonal therapy such as abiraterone acetate, enzalutamide, and/or apalutamide.

Somatic testing was gathered using Guardant360 (Redwood City, CA), and germline data was gathered primarily using Invitae Multi-Cancer Panel (San Francisco, CA). Other sources of germline data included Common Hereditary Cancers Panel (San Francisco, CA), Prostate Cancer Panel (Gaithersburg, MD), and Ambry Genetics/CancerNext + RNA (Aliso Viejo, CA). All genomic tests were obtained as system on chip through Clinical Laboratory Improvement Amendments certified labs.

The primary objective was to describe the baseline patient and disease characteristics and treatment patterns of mHSPC Black male patients. Variables of interest included Gleason score, pathology, prostate-specific antigen (PSA) level, age at diagnosis, and disease volume at time of stage 4 diagnosis. “Disease volume” was defined by CHAARTED criteria (presence of 4 or more bone metastases with at least one outside the pelvis and spine, and/or visceral metastases).^10^ “High-risk” status was defined by LATITUDE criteria (presence of at least 2 of the following: 3 or more bone metastases, visceral metastases, Gleason 8 or higher).^12^ Treatment for mHSPC and subsequent lines of treatment for CRPC (when available) were captured.

Secondary objectives included time to CRPC, overall survival, PSA response, as well as the germline and somatic data. Time to CRPC was defined as the time to PSA progression, radiographic evidence of disease progression, or time to clinical progression per local investigator’s assessment. Overall survival was calculated using time from diagnosis to death or last follow-up. Undetectable PSA was defined as <0.1 ng/mL. For germline data, pathogenic alterations and variants of uncertain significance (VUS) were collected. Somatic data were examined for pathogenic alterations (both the gene and the genomic alteration). Data were also collected relating to mortality, first-line treatment and number of subsequent treatments for CRPC, and the need for palliative radiation to metastatic sites.

### Statistical Analysis

Microsoft Excel and SPSS (IBM Corp©, version 23.0.0.0) were used to calculate summary statistics for baseline characteristics, treatment, and genetic data. Time-to-event analysis was calculated using the Kaplan-Meier estimator. PSA progression, scan progression, or clinical progression, time to CRPC, and survival data were calculated based on dates given by the collecting site.

## Results

### Baseline Characteristics

A total of 107 patients (median age 64 years, range 41-84) were included in the final analysis. The median follow-up time from stage 4 diagnosis was 2.2 years (IQR 1.5 years to 3.6 years). Sixty-three patients (59%) had Gleason 8-10, 66 patients (62%) had newly diagnosed metastatic disease, and the median initial PSA at diagnosis was 97.8 ng/mL (range 0.9-650). Sixty-nine patients (66%) had high-volume disease based on CHAARTED criteria, including 17 (16%) of patients with visceral disease and 60 patients (58%) with high-risk disease (LATITUDE criteria). There were 2 cases of adenocarcinoma with neuroendocrine features and one pure small cell tumor. Baseline characteristics are described in Table 1.

**Table 1.** Patient characteristics of 107 Black men with mHSPC.

Baseline characteristics	*N* (%)
Median age (range)	64 years (41-84)
Median PSA at stage 4 (range)	97.8 ng/dL (0.9->7650)
Unknown	8 (7)
Gleason Score at diagnosis	
8-10	63 (59)
0-7	29 (27)
Unknown	15 (14)
Disease status at time of HSPC (*N* = 107)	
Newly diagnosed (de novo)	66 (62)
Recurrent	41 (38)
Unknown	0 (0)
Prior definitive treatment for local disease (*N* = 41)	
Surgery	20 (49)
Radiation	29 (71)
ADT	14 (34)
Orchiectomy	2 (5)
Unknown	2 (5)
Pathology	
Adenocarcinoma	91 (85)
Adenoc. with neuroendocrine features	2 (2)
Small cell	1 (1)
Missing data	13 (12)
Disease volume	
*>*3 bone metastases	70 (66)
Lymph node metastases	61 (58)
Visceral disease	17 (16)
High-volume per CHAARTED^a^	69 (66)
High-risk disease per LATITUDE^b^	60 (58)

^a^High-volume per CHAARTED defined as presence of visceral metastasis or ≥4 bone lesions with ≥1 beyond the vertebral bodies and pelvis.

^b^High-risk disease per LATITUDE defined as meeting 2 of 3 criteria: Gleason score of ≥8, ≥3 bone lesions, presence of visceral metastases.

Abbreviations: ADT, androgen deprivation therapy; HSPC, hormone sensitive prostate cancer; PSA, prostate-specific antigen.

### Clinical Outcomes

We evaluated the clinical outcomes on 106 patients (1 patient was excluded due to missing data). Overall, 27% of patients (*N* = 29) were treated with ADT with or without first-generation anti-androgens; most of the cases (88%) were prior to the year 2015. Treatment intensification with either chemotherapy or novel hormonal therapies was used in most cases (73%, *N* = 77); 20% received a chemotherapy-based regimen. Eight patients received triplet therapy (8%) with ADT plus docetaxel and abiraterone (*N* = 2) or enzalutamide (*N* = 6), and 1 patient was treated with carboplatin plus etoposide plus ADT. The treatment intensification cohort had a significantly higher number of patients with de novo metastatic disease (*P* = .038), a numerically higher number of high-volume disease (*P* = .094) and higher initial median PSA at time of advanced disease (PSA = 435 vs 64.8 ng/mL) compared with the ADT cohort.

Eleven patients (10%) received radiation to the prostate gland after diagnosis of stage 4 disease, and 5 patients had low-volume disease. Undetectable PSA was achieved in 42 patients (40%), more commonly with abiraterone acetate (*N* = 24/40) and enzalutamide (*N* = 3/5). At time of cut-off, 57 patients (54%) developed CRPC, and the median time to CRPC was 25.3 months (95% CI, 21.4-29.3). Patients treated with ADT with or without first-generation anti-androgens had a median time of CRPC of 46.3 months (18.9-73.7). Those in the ADT ± anti-androgen group were less likely to present with de novo disease than patients in the treatment intensification group (*P* = .006) but not statistically less likely to have high-risk disease (*P* = .527) or high-volume disease (*P* = .102).

Patients with either high-volume disease (CHAARTED criteria), with treatment intensification, or with detectable nadir PSA while on HSPC therapy were associated with a significantly shorter time to CRPC (18.9 vs 99.6 months, *P < .*001; 23.3 vs 46.3 months, *P =* .031; 13.5 vs 46.3 months, *P <* .001, respectively). Patients with visceral disease also had shorter time to CRPC although this was not statistically significant (28.0 vs 19.8 months, *P* = .079). Neither newly diagnosed metastatic disease (22.6 vs 33.1 months, *P =* .096) nor Gleason score 8-10 (24.0 vs 26.0 months, *P =* NS) were associated with shorter time to CRPC.

### Subsequent Treatments for CRPC and Overall Survival

**Figure 1.**
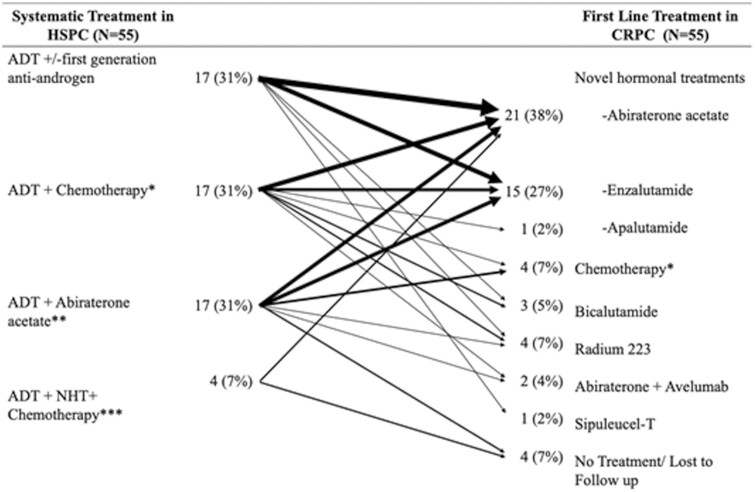
Treatment sequence for patients who developed CRPC (*N* = 55). Abbreviations: ADT: androgen deprivation therapy; CRPC: castrate-resistant prostate cancer; HSPC: hormone sensitive prostate cancer; NHT: novel hormonal therapy. Note: arrows’ width represents the frequency of each treatment sequence. ∗One patient (*N* = 1) underwent docetaxel therapy in HSPC and carboplatin plus cabazitaxel combination therapy in CRPC; ∗∗*N* = 5 patients were treated with abiraterone acetate in both the HSPC and CRPC stages. Four patients underwent prednisone/ dexamethasone switch at time of CRPC. One patient discontinued abiraterone acetate due to adverse effects but resumed same therapy at time of CRPC. ∗∗∗Androgen deprivation therapy plus docetaxel plus enzalutamide (*N* = 3) and ADT plus docetaxel plus abiraterone acetate (*N* = 1). The latter patient received no treatment in the CRPC stage.

Among the 55 patients who developed CRPC with data available, the subsequent therapy was most commonly abiraterone acetate (38%), followed by enzalutamide (27%), chemotherapy (7%), Radium-223 (7%), and no subsequent treatment (7%). Of the 17 patients who underwent treatment with abiraterone originally, 10 (59%) were treated with another novel hormonal therapy in the CRPC stage. Of these 10, half were continued on a form of abiraterone and half were switched to enzalutamide. Of the 4 patients who underwent treatment with ADT + NHT + chemotherapy in the HSPC stage, 2 were switched to an NHT. In both cases, patients went from ADT + Enzalutamide + chemotherapy in the HSPC stage to Abiraterone in the CRPC stage. Patients underwent a median of 2 lines (range 0-7) of treatment for CRPC (Fig. 1).

The 2-year survival rate from time of initial diagnosis of mHSPC was 93.3%. The estimated median overall survival (OS) in this cohort was 74.9 months (95% CI, 68.7-81.0; Table 2).

**Table 2.** Clinical outcomes of the cohort overall and grouped by mHSPC treatment regimen (*N* = 106).

Treatment received	Total (%)	De novo metastatic disease (%)	High-volume (CHAARTED)(%)	Undetectable PSA (%)	CRPC (%)	Median time to CRPC, months, (95%CI)
Overall	106 (100)	66 (62.3)	68 (64.2)	42 (39.6)	57 (53.8)	25.4 (20.3-30.4)
# Unknown		0 (0)	2 (1.9)	13 (12.3)	4 (3.8)	
ADT ± first-generation anti-androgen	29 (27.4)	12 (41.4)	15 (51.7)	7 (24.1)	18 (62.1)	46.3 (18.9-73.7)
Treatment Intensification^a^	77 (72.6)	54 (70.1)	53 (68.8)	35 (45.5)	39(50.6)	23.4 (18.6-28.2)
|ADT plus chemotherapy^b^	21 (19.8)	16 (76.2)	18 (85.7)	4 (19.0)	17 (81.0)	16.2 (12.3-20.1)
|Novel hormonal therapy	48 (45.3)	15 (31.3)	29 (60.4)	27(56.3)	18 (37.5)	26.6 (16.8-36.4)
ADT plus abiraterone acetate	40 (37.7)	27 (67.5)	25 (62.5)	24 (60.0)	17 (42.5)	28.1 (17.1-39.0)
ADT plus enzalutamide	5 (4.7)	3 (60.0)	1 (20.0)	3 (60.0)	1 (20.0)	22.6 (3.1-42.1)
ADT plus apalutamide	3 (2.8)	3 (100)	3 (100)	0 (0)	0 (0)	—
|ADT plus chemotherapy plus NHT^c^	8 (7.5)	5 (62.5)	6 (75.0)	4 (50.0)	4 (50.0)	17.8 (10.3-25.3)

^a^Includes ADT plus chemotherapy and/or novel hormonal therapy.

^b^Includes: *N* = 20 docetaxel, *N* = 1 carboplatin plus etoposide.

^c^Includes: *N* = 6 ADT + enzalutamide + docetaxel, *N* = 2 ADT + abiraterone acetate + docetaxel.

Abbreviations: ADT, androgen deprivation therapy; CRPC, castrate resistant prostate cancer.

### Genomic Germline and Somatic Data

**Figure 2.**
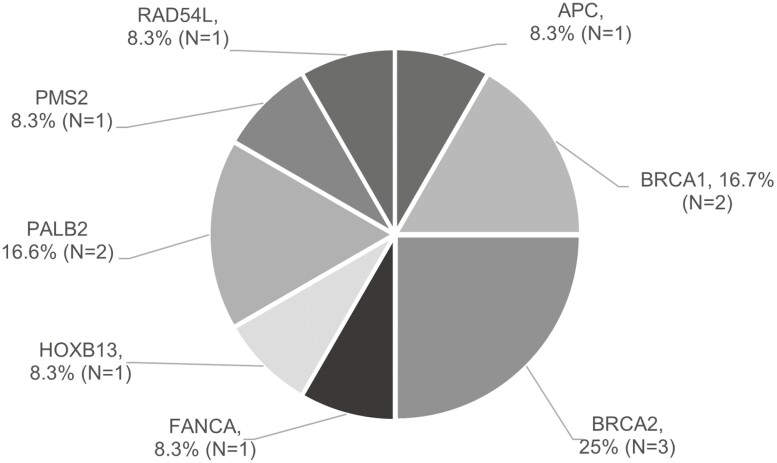
Pathogenic and likely pathogenic germline mutations (*N* = 12) in mHSPC patients with available germline test (*N* = 96).

Most patients (78%) had genetic testing done in the HSPC stage, a median of 14 days after stage 4 diagnosis (range 0 days and 11 years). Germline data were available for 96 patients (90%). Among these, 12% tested positive for at least 1 pathogenic/likely pathogenic mutation in a prostate cancer genetic panel. *BRCA2* was found most frequently (*N* = 3) followed by *BRCA1* (*N* = 2) and *PALB2* (*N* = 2). Other pathogenic mutations included *RAD54L*, *PMS2*, *HOXB13*, *FANCA*, and *APC* (Fig. 2). Among VUS, the most frequently seen were in *RECQL4*, *ATM*, and *POLE* (*N* = 6).

Additionally, somatic testing was available for 50 patients (47%) after excluding 2 due to insufficient quality. Next generation sequencing results included tissue sample (40%), ctDNA (52%), and both in 3 patients. This most often revealed at least one pathogenic genomic alteration (median = 1), most commonly *TP53* (24%), *CDK12* (12%), *TMPRSS2-ERG* fusion (10%), and *SPOP* (8%) (Table 3). Patients with *TP53* mutated tumors were associated with a higher number of de novo disease, high-volume disease, development of CRPC, and inferior PSA response rate, although without statistical significance (Table 4).

**Table 3.** Somatic alterations in mHSPC patients with somatic available testing performed (*N* = 50), as well as frequency and assay used.

Somatic alteration	*N* (%)	Next-generation sequencing assay (*N*)
None	14 (28%)	
TP53		
*Q167; splice site SNV; R267fs; c.578A>G p.H193R; c.743G>A; p.R248Q; R175H, N239S + R248Q; H179Q; N131 del; R342; K132R; 1 unspecified*	12 (24%)	Tissue (4); ctDNA (7); unknown (1)
CDK12		
*S133fs+p.S133fs; p.D121fs+p.M972fs; c.1086T>G p.Y362; 162delG+1066A>T; 2 unspecified*	6 (12%)	Tissue (3); ctDNA (2); Both (1)
TMPRSS2		
*4 ERG-fusion; 1 unspecified*	5 (10%)	Tissue (3); ctDNA (1); Both (1)
SPOP		
*p.F133L; p.F125V; c.305T>G p.F102C; W131R*	4 (8%)	Tissue (4)
AR		
*1 amplification; 2 AR-V7*	3 (6%)	Tissue (2); ctDNA (1)
CTNNB1		
*D32Y; p.D32N; S45P-subclonal*	3 (6%)	Tissue (1); ctDNA (2)
PIK3CA *(H1047R; amplification)*	2 (4%)	Tissue (1); ctDNA (1)
CCNE1 amplification	2 (4%)	ctDNA (2)
PTEN*(130Q; deletion)*	2 (4%)	Tissue (1); ctDNA (1)
TMB (high)	1 (2)	Tissue (1)

ctDNA (circulating tumor DNA): Guardant 360, Strata; Tissue: Foundation One, Caris. TMB (tumor mutational burden); high defined as ≥10 Mb.

**Table 4.** Outcomes data based on the 2 most common somatic alterations (>10%). For every outcome, patients with the somatic alteration of interest are compared to wild type, or patients with somatic data available but without the somatic alteration of interest (*N* = 50).

Somatic alteration		De novo metastatic disease (%)		High-volume (CHAARTED) (%)		Undetectable PSA (%)		CRPC (%)	
TP53 (*N* = 12)	Mutant	67%	*P* = .59	82%	*P* = .83	27%	*P* = .16	50%	*P* = .30
								30%	
	WT	58%		53%		51%			
CDK12 (*N* = 6)	Mutant	67%	*P* = .30	50%	*P* = .53	50%	*P* = .82	50%	*P* = .45
	WT	59%		60%		45%		34%	

WT: wild type (somatic data available and mutation of interest is absent); CRPC: castrate resistant prostate cancer.

## Discussion

This study evaluated the outcomes of Black patients with mHSPC from different academic institutions with an interest in racial disparities. This study focused exclusively on patients with available genomic data because we were interested in assessing the molecular profile in this setting. To our knowledge, this is the largest cohort of Black patients reported since the incorporation of chemotherapy and novel hormonal therapies as the standard of care for patients with mHSPC. We reported favorable clinical outcomes in terms of time to CRPC and OS including patients treated with ADT with or without first generation anti-androgens, and we noted a heterogenous management of this patient population in terms of systemic therapies used and the type and panel of the genomic testing performed.

In mHSPC, Black men represented <5% of all patients enrolled in the randomized phase III trials in this setting, except for the SWOG S1216 study which enrolled 10% of Black patients.^10,16-18,27^ When compared with the trial population included in those studies, our cohort consisted of patients with a lower median age and similar rate of Gleason score 8-10 at time of mHSPC, but with a higher proportion of high-volume disease, an increased number of visceral metastases, and a higher initial PSA at time of stage 4.^10,12,14-16,27^

In our dataset, only a small fraction of patients (19%) who were treated for mHSPC after 2015 were offered ADT alone. However, real-world data suggest the opposite in that most patients with mHSPC—and more Black than White patients—are currently receiving ADT regimens without treatment intensification.^28^ Of note, the time to CRPC of almost 4 years in this ADT subgroup was approximately twice as long as the observed progression-free survival in the control arms of all phase III trials.^10-16,27^ This may be skewed as patients with recurrent (non-de novo) were overrepresented in this cohort. The time to CRPC in this study was defined by PSA, radiographic, or clinical progression. Most patients met serologic criteria, which typically precedes the time of radiographic progression in several months.^29^ Thus, this difference in time to CRPC seems to suggest that Black patients achieve better treatment outcomes, requiring further validation.

Notably, the median time to CRPC was much shorter in patients who received treatment intensification compared with ADT with and without first-generation anti-androgen, which might be explained by the over-representation of high-volume disease in this first group. In fact, the factors associated with shorter time to CRPC such as disease volume and detectable nadir PSA have been reported in previous studies and support these findings.^30-34^ Additionally, the differences observed by type of agent (chemotherapy vs novel hormonal therapy) seem to favor the addition of a novel hormonal agent in this setting, but more studies are needed to confirm these findings.^35,36^

The access to emergent life-prolonging therapies during advanced disease significantly impacts the outcomes of prostate cancer patients. We observed an encouraging 2-year OS (>90%) that compares favorably with trial data with any combination regimen in this space. Additionally, we confirmed the access to subsequent effective and emergent therapies of patients who progressed to CRPC. Considering the ADT-only subgroup of patients, the relatively fewer number of patients with newly diagnosed and low volume disease might help to explain the long time to CRPC of almost 4 years. A longer follow-up is required to confirm these encouraging results.

Almost all our patients (90%) had germline testing. Germline mutations were found in a subset of patients, with many that involved DNA repair genes; this has also been previously reported.^25^ The *BRCA2* germline mutation was found to be the most prevalent DNA repair gene alteration, which was similar to a large germline study involving mostly White men with metastatic prostate cancer.^37^ Poly(ADP-ribose) polymerase inhibitors have demonstrated success in treating patients with cancer with germline *BRCA* mutations, with olaparib and rucaparib now FDA-approved treatments in the setting of CRPC with these mutations.^38,39^ Black patients with these germline mutations discovered in the mHSPC setting may warrant targeted therapy, and there is an ongoing study testing this hypothesis.^40^

Fewer patients had somatic testing done, using 4 different assays. Liquid biopsy was used more frequently than tissue-based sequencing test, which is often due to the lack of tissue samples available. The relatively low number of genomic alterations, including in the *AR* gene, is consistent with low exposure to systemic therapies including castration.^41^ In this sample, some patients underwent genetic testing before exposure to systemic therapies, but others received testing later during treatment. The high frequency of *CDK12* genomic alterations is again confirmed in this patient population as shown by our group recently,^42^ with prognostic and therapeutic implications at time of progression to CRPC.^43^*TP53* was the most common somatic alteration detected; this is found in over half of patients with CRPC,^44^ and it may contribute to the increased aggressiveness of prostate cancer in Black men. In our cohort, *TP53* mutated tumors had a numerically higher number of de novo disease, high-volume disease, development of CRPC, and PSA nonresponse, although without statistical significance.

Published studies have revealed that Black patients are diagnosed at a younger age, have more aggressive disease, yet clinical outcomes in this cohort do not appear to be inferior.^45-47^ Prospective studies with approved therapies for metastatic disease have suggested Black patients may respond better than White patients, as suggested with docetaxel (meta-analysis of nine phase II/III trials)^22^ and sipuleucel-T (PROCEED, NCT01306890).^24^

These findings emphasize the increasing effort to investigate racial disparities in prostate cancer and several clinical trials and registries are actively enrolling Black patients. As an example, the PANTHER trial (NCT03098836) is testing the combination of abiraterone acetate with apalutamide in 2 cohorts of patients with CRPC including Black men. Beyond clinical trial concepts, large national and international consortiums such as the PROMISE consortium^48^ and the IRONMAN registry (NCT03151629) with the Diversity Working Group^49^ are examples of the efforts to further capture clinical outcomes, epidemiological data, and molecular profile of different racial and ethnic groups.

The limitations of this study include its retrospective nature and the relative short follow-up to explore treatment sequencing and outcomes at time of progression to CRPC. There was a selection bias since all patients were included from academic cancer centers in the US, most of which are in urban areas which may not be generalizable to patients in more rural environments, or environments outside of the USA. Furthermore, this study identified patients with genetic testing data available (despite the short interval between the diagnosis of stage 4 disease and genetic test) which might have contributed to the selection bias. Although the majority of patients received genetic testing close to initial stage 4 diagnosis, not every patient received somatic testing at the same time; therefore, there is potential immortal time bias as patients with less severe disease may have lived longer and been able to receive somatic testing later in the disease course. African ancestry was not confirmed with genetic testing, which may create heterogeneity of the genomic samples and therefore skew or dilute the genetic results. Despite being one of the largest cohorts in this patient population, the genomic data are limited by the scope of and differences between the gene panels of choice, which limits definitive conclusions.

## Conclusions

In this cohort of Black patients with HSPC, the observed clinical outcomes were very favorable with ADT-based regimens. The genomic testing revealed different germline and somatic mutational with significant impact in the management of these patients.

## Data Availability

The data underlying this article will be shared on reasonable request to the corresponding author.
